# Migration and section of esophageal thermometer in laparoscopic sleeve gastrectomy: Clinical case of a preventable complication

**DOI:** 10.1016/j.ijscr.2024.110399

**Published:** 2024-10-02

**Authors:** Antonio de Jesús González-Luna, Marco Antonio Castellanos-López, Matthew Abel Juárez-Mora, Karyme Naomy González-Jiménez, Quitzia Libertad Torres-Salazar

**Affiliations:** a Regional Hospital “Dr. Valentín Gómez Farías”, Institute of Security and Social Services for State Workers, Mexico; b National Polytechnic Institute, Mexico; c Autonomous University of Guadalajara, Mexico; d Juárez University of the State of Durango, Mexico

**Keywords:** Laparoscopic sleeve gastrectomy, temperature probe, complications

## Abstract

**Introduction and importance:**

Laparoscopic sleeve gastrectomy is a prevalent bariatric surgery for managing morbid obesity. Despite its efficacy, complications can arise, particularly from intraoperative devices such as esophageal thermometers and orogastric tubes. These devices, if misplaced or inadequately monitored, can migrate and become entrapped or sectioned during surgery, leading to significant morbidity.

**Case presentation:**

A 49-year-old female with morbid obesity underwent LSG. During surgery, an esophageal thermometer migrated into the stomach and was inadvertently sectioned during the stapling process. This required additional surgical intervention to remove the severed segments and repair the damage. Postoperative recovery was uneventful after corrective measures were taken.

**Clinical discussion:**

This case underscores the critical importance of ensuring the proper placement and continuous monitoring of intraoperative devices, such as esophageal thermometers, to prevent similar preventable complications in future surgical procedures.

**Conclusion:**

The reviewed cases demonstrate that complications from intraoperative device migration and entrapment during bariatric surgery, while rare, are significant and preventable. Adherence to strict protocols, continuous device monitoring, and enhanced team communication are essential to improve patient safety and surgical outcomes. Implementing these measures can prevent avoidable complications and enhance the efficacy of bariatric surgeries.

**Evidence based medicine ranking:**

Level IV.

## Highlights

• Complications from laparoscopic sleeve gastrectomy can arise due to misplacement of intraoperative devices.
• In this case, an esophageal thermometer migrated into the stomach and was sectioned, requiring further surgery.
• This highlights the importance of correct device placement and continuous monitoring to prevent complications
• Adherence to strict protocols and vigilant monitoring are essential for improving patient safety and surgical outcomes.
• The patient experienced a successful postoperative recovery with no further complications.

## 1 Introduction

Laparoscopic sleeve gastrectomy (LSG) is a widely accepted bariatric procedure for the management of morbid obesity [1]. The surgery involves the removal of a large portion of the stomach, resulting in a tubular gastric sleeve, which significantly restricts food intake and induces weight loss [2]. Despite the efficacy and safety profile of LSG, it is not devoid of complications. Common complications include staple line leaks, hemorrhage, and strictures [3]. However, iatrogenic complications related to the use of intraoperative devices are less frequently reported.

Esophageal thermometers are routinely used during surgeries to monitor core body temperature accurately [4], indeed National Institute of Clinical Excellence recommends continuous monitoring of core body temperature using an insertable temperature probe is part of monitoring in major operations [5]. These devices are designed to remain within the esophagus, and their migration into the stomach is uncommon. However, if such migration occurs, it can lead to inadvertent sectioning or damage during the surgical procedure, resulting in potential complications.

In this clinical case, we present an unusual complication where an esophageal thermometer migrated into the stomach and was subsequently sectioned during the stapling process of an LSG. This incident highlights the importance of proper placement and continuous monitoring of intraoperative devices to prevent such avoidable complications. Through this case, we aim to discuss the steps taken to address the complication, the preventative measures that can be implemented, and the implications for surgical practice. This report follows the SCARE criteria [6].

## 2 Clinical case description

A 49-year-old female patient with a history of morbid obesity (BMI: 54.65 kg/m^2^), type 2 diabetes mellitus, hypertension, and primary hypothyroidism presented for a laparoscopic sleeve gastrectomy and prophylactic cholecystectomy due to incidental cholelithiasis. The patient had a significant history of obesity-related comorbidities and had previously attempted weight management through non-surgical means without success.

During the preoperative evaluation, the patient's physical examination and laboratory results were within acceptable ranges for surgery. The patient was taken to the operating room, where standard aseptic and antiseptic protocols were followed. Under general anesthesia, the patient was placed in the French position, and sterile drapes were applied.

A pneumoperitoneum was established, and trocars were strategically placed. The surgical team proceeded with the dissection of gastroepiploic vessels using harmonic energy from 5 cm proximal to the pylorus to the gastric fundus. Calibration of the gastric sleeve was performed with an orogastric tube.

**Fig. 1:**
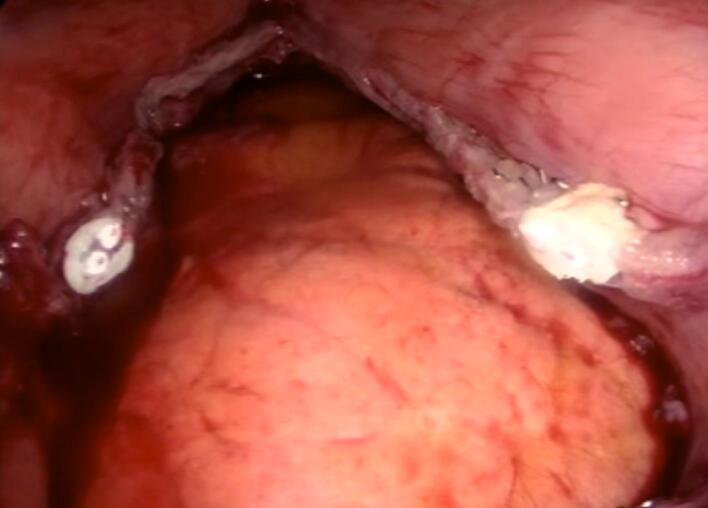
The esophageal thermometer is observed to be transected, with both internal ends visibly severed.

**Fig. 2:**
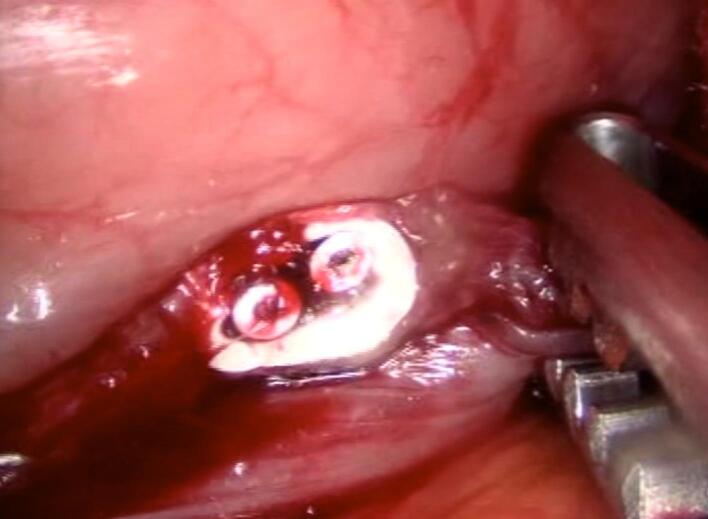
The esophageal thermometer section is observed at the stapling line.

Unexpectedly, during the stapling of the stomach using a Covidien stapler with purple cartridges, the esophageal thermometer, which had migrated from its proper position in the esophagus to the stomach, was sectioned into two segments (Fig. 1). Both internal ends of the thermometer were visualized within the stomach (Fig. 2). This migration is atypical, as esophageal thermometers are intended to remain in the esophagus, as recommended by the National Institute for Health and Care Excellence (NICE) for continuous core body temperature monitoring in major surgeries.

**Fig. 3:**
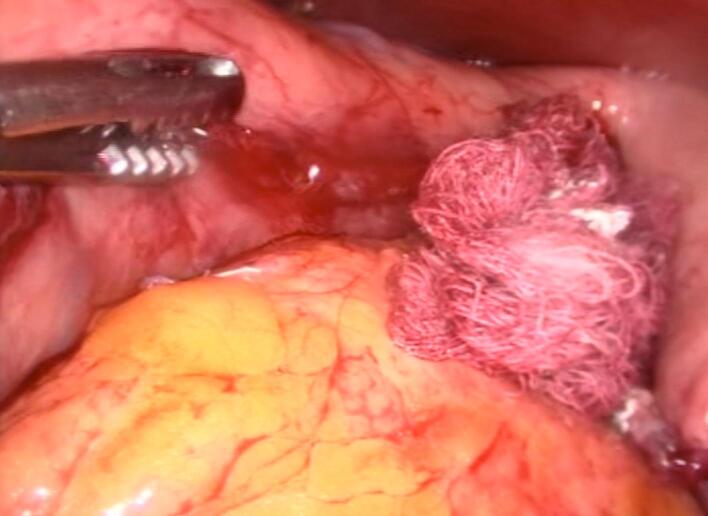
The thermometer cable was removed using Maryland scissors and external traction via the oral route. Gauze was introduced (as shown in the image) to clean the defect area.

**Fig. 4:**
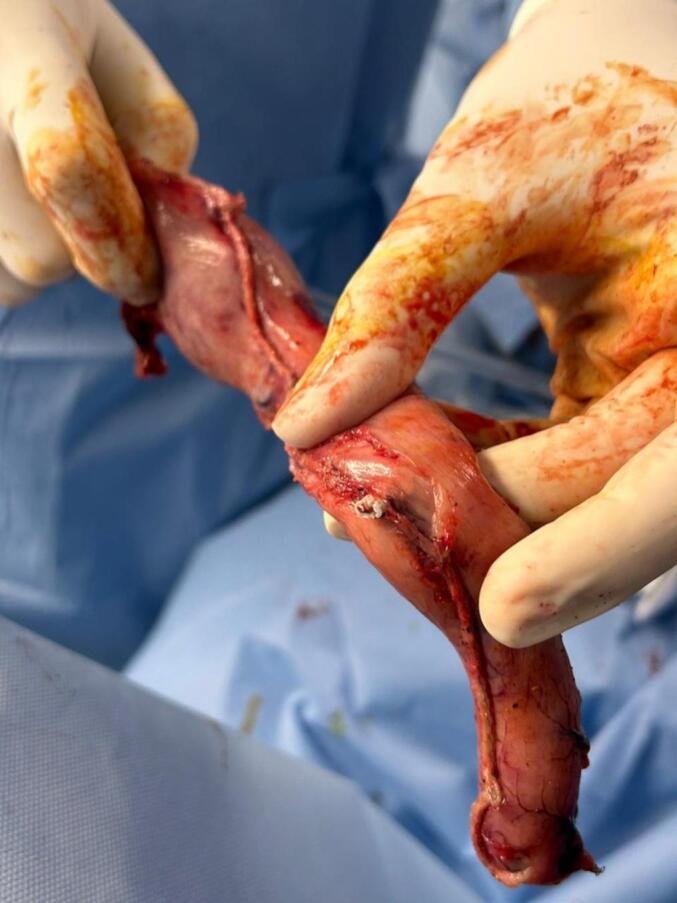
The thermometer cable (severed internal segment) was visualized in the extracted gastric segment.

The surgical team completed the stapling of the gastric sleeve and carefully removed the sectioned thermometer segments using a Maryland forceps (Fig. 3). The site where the thermometer was sectioned was reinforced with continuous sutures using PDS 2–0 and Lembert sutures for additional security. The entire staple line was further reinforced with Monocryl 3–0 sutures (Fig. 4).

**Fig. 5:**
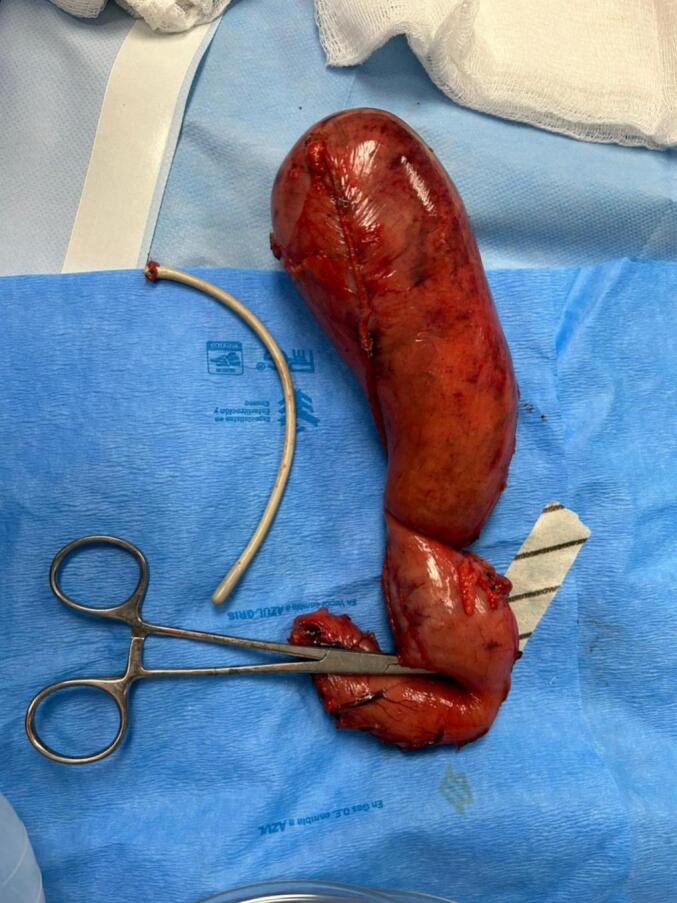
The thermometer cable was visualized in the excised gastric segment.

**Fig. 6:**
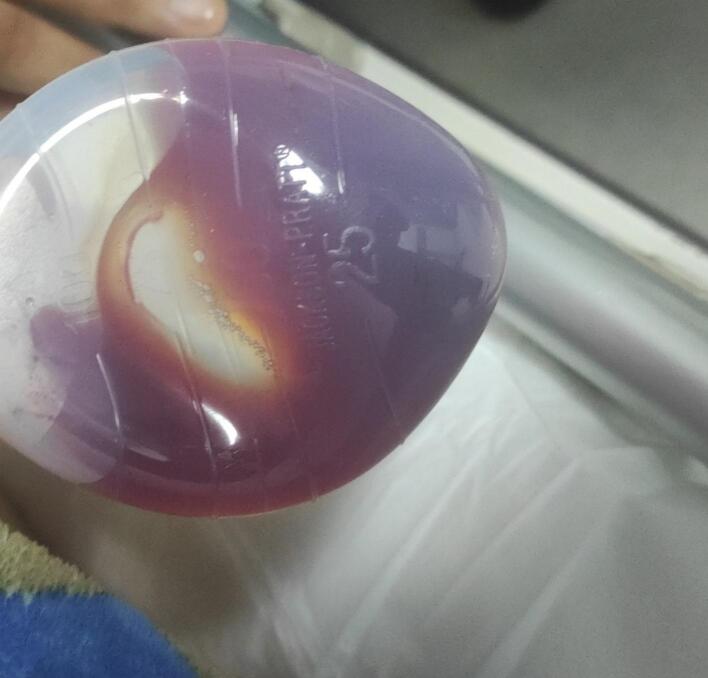
A new methylene blue test was performed the following day, showing no evidence of leakage in the Jackson-Pratt drain.

A methylene blue leak test was performed, which showed no evidence of leakage. The surgery proceeded with the laparoscopic cholecystectomy without further complications. A Jackson-Pratt drain was placed near the surgical site for postoperative monitoring (Fig. 5, Fig. 6). The patient was hemodynamically stable postoperatively and was transferred to the recovery unit. Subsequent evaluations, including esophagogastroduodenoscopy and fluoroscopic examination, confirmed the absence of leaks. The patient was discharged in stable condition with specific dietary guidelines and instructions for drain care.

## 3 Discussion

The case presented here involves the migration and section of an esophageal thermometer during LSG, highlighting a preventable complication. This situation is contrasted with similar cases reported in the literature to underscore the importance of vigilance and adherence to safety protocols during such procedures.

In our case, the esophageal thermometer, initially placed correctly, migrated into the stomach and was inadvertently sectioned during the stapling process. This necessitated additional surgical intervention to remove the severed segments and repair the damage. This case underscores the critical need for continuous monitoring and proper placement of intraoperative devices to prevent such incidents.

Comparatively, Wass et al. reported a case of nasopharyngeal temperature probe entrapment during a revision laparoscopic Nissen fundoplication. In this instance, the temperature probe, initially placed in the nasopharynx, was inadvertently entrained into the esophagus during the insertion of an esophageal bougie, leading to its entrapment by a fundoplication ligature [7]. The complication was detected at the end of the surgery when the anesthesia provider could not remove the probe. The surgical team had to re-establish pneumoperitoneum, take down the fundoplication, and remove the probe before revising the fundoplication without further incident.

Similarly, Raghavendra and Balupuri reported a case of temperature probe entrapment during LSG, where the probe migrated into the stomach and was stapled across the gastric sleeve. This led to postoperative staple line dehiscence and the need for emergency laparotomy and upper GI endoscopy to remove the entrapped probe and repair the staple line. This case also highlighted the severe postoperative complications, including acute respiratory distress syndrome (ARDS) and the need for prolonged intensive care [8].

Yanovski et al. described another case where a nasally inserted thermometer probe was entrapped and cut during gastric stapling in LSG. The probe, inserted via the right nostril and positioned in the hypopharynx, was inadvertently pushed into the surgical field by the bougie, leading to its sectioning into three parts during the stapling process. Prompt detection and corrective measures, including loosening sutures and removing the probe segments, prevented further complications. This case emphasized the importance of correct initial placement and constant monitoring of the probe's position during surgery [9].

Çalıkoğlu et al. conducted a systematic review and reported on the inadvertent stapling of orogastric tubes (OGTs) during bariatric surgery. Their findings revealed that OGTs, temperature probes, and bougies are frequently entrapped and stapled, leading to increased risks of leaks, reoperations, and even mortality. The review emphasized the need for strict protocols to ensure the removal of OGTs before stapling and recommended increased cooperation between anesthesiologists and surgeons to prevent such complications [10].

These cases collectively emphasize the preventable nature of such complications through better initial placement, vigilant monitoring, and effective communication between surgical and anesthetic teams. The use of alternative monitoring methods, such as bladder probes for core temperature monitoring in patients with indwelling urinary catheters, and strict adherence to protocols to ensure that all foreign bodies are accounted for before critical surgical steps, can significantly mitigate these risks. Implementing these measures can enhance patient safety and improve surgical outcomes in bariatric and other upper gastrointestinal surgeries.

## 4 Conclusion

Both the clinical case presented and the reviewed cases of intraoperative entrapment and sectioning of esophageal and nasopharyngeal temperature probes, as well as orogastric tubes during laparoscopic bariatric surgeries, underscore the critical need for vigilance and strict protocols. These incidents, while rare, can lead to significant complications, including leaks, reoperations, and severe postoperative morbidity. The key to preventing such events lies in ensuring the proper placement and continuous monitoring of all intraoperative devices, enhanced communication between surgical and anesthetic teams, and adherence to stringent safety protocols. These measures are essential for improving patient safety and surgical outcomes in bariatric and other upper gastrointestinal procedures.

## Consent

Written informed consent was obtained from the patient for publication and any accompanying images. A copy of the written consent is available for review by the Editor-in-Chief of this journal on request.

## Ethical approval

The present study is the presentation of a clinical case, we point out that in our institution it is not necessary to be submitted to or approved by an ethics committee, the host institution to which we belong corresponds to the Hospital del Instituto de Seguridad y Servicios Sociales para lo Trabajadores del Estado, de Zapopan in the State of Jalisco.

## Funding

Nothing to declare.

## Author contribution

AJGL - Diagnosis and follow-up and Surgical approach plan.

MACL- File tracking and documentation.

MAJM- Bibliographic review.

KNGJ- Data analysis.

TSQL- Article redaction.

## Guarantor

Quitzia Libertad Torres Salazar.

## Research registration number

N/A.

## Conflict of interest statement

Nothing to declare.
